# Effects of the Dietary Inclusion of *Allium mongolicum* Regel Extract on Serum Index and Meat Quality in Small-Tailed Han Sheep

**DOI:** 10.3390/ani13010110

**Published:** 2022-12-27

**Authors:** Yabo Zhao, Yanmei Zhang, Chen Bai, Changjin Ao, Saruli Qi, Qina Cao, Khas Erdene

**Affiliations:** Key Laboratory of Animal Feed and Nutrition of Inner Mongolia Autonomous Region, College of Animal Science, Inner Mongolia Agricultural University, Hohhot 010018, China

**Keywords:** natural extract, body health, meat physicochemical characteristics, amino acids, fatty acids

## Abstract

**Simple Summary:**

*Allium mongolicum* Regel, a typical herb of the Allium family, is rich in a variety of active ingredients, such as flavonoids, essential oils, and polysaccharides. Our previous studies have reported that the dietary supplementation of lambs’ diets with flavonoids from *A. mongolicum* Regel could promote growth performance and improve the meat quality and mutton flavor of lambs. However, the flavonoid extraction process is complicated, time-consuming, and costly, making it difficult to apply in practical production. *A. mongolicum* Regel ethanol extract (AME) is easier to obtain and less expensive than flavonoids. AME also contains many bioactive compounds. In the present study, we investigated the effects of AME on the serum index and muscle physicochemical characteristics, as well as the amino acid and fatty acid composition in Small-tailed Han sheep. The results indicated that AME could regulate lipid metabolism and promote the deposition of free amino acids and unsaturated fatty acids in the longissimus dorsi muscle of lambs. These findings suggested that AME could be used instead of flavonoids to improve the body health and meat quality of lambs in practical production.

**Abstract:**

The aim of this study was to evaluate the effects of *Allium mongolicum* Regel ethanol extract (AME) on the serum index and meat quality of lambs. A total of 30 male Small-tailed Han sheep (3 months old) with an average weight of 33.60 ± 1.23 kg were divided randomly into one of two groups: the control group (CON) was offered a basal diet, and the AME group was offered a basal diet with supplementation 2.8 g·lamb^−1^·day^−1^ AME. The trial lasted for 75 days. AME supplementation significantly decreased the concentration of triglyceride and total cholesterol (*p* < 0.05), and tended to lower the concentration of non-esterified fatty acids (0.05 < *p* < 0.1), but significantly increased the concentration of high-density lipoprotein, leptin, and insulin (*p* < 0.05) in the serum of lambs. AME also decreased cooking losses and shear force and increased the content of intramuscular fat in the longissimus dorsi (LD) muscle of lambs (*p* < 0.05). In addition, there was no difference in the composition of hydrolyzed protein amino acids in the LD muscle among treatments (*p* > 0.05). However, AME changed the composition of free amino acids and promoted MUFA and PUFA deposition in the LD muscle of the lambs. These findings indicate that a diet supplemented with AME may improve the lipid metabolic capacity and meat quality of lambs.

## 1. Introduction

Mutton is rich in protein, minerals, vitamins, and other nutrients. In the last two decades, with the improvement in the living standards of the population, there has been an increasing demand for mutton or mutton products in China [1]. To meet the market demand, the feeding regime of lambs had changed from extensive grazing to feedlot feeding [2], which provides constant dietary supplements to increase the growth rate of lambs, resulting in carcass characteristics that meet market demand. This feeding regime can constantly supply mutton production for the market demand and simultaneously affect the body’s health status, meat quality, and mutton flavor [3,4]. The use of drugs can maintain the healthy state of lambs, but there are also problems associated with the use of drugs, such as drug resistance and toxin residuals in meat, which seriously affect the safety of the meat products. Therefore, there is a growing interest in finding natural additives to replace traditional drugs. In recent years, many studies have reported that natural plants or their extracts can efficiently improve meat quality and mutton flavor of lambs grown under feedlot feeding regimes [5,6]. The herdsmen observed that lambs consuming *Allium mongolicum* Regel experienced fewer diseases, grew faster, and produced meat with light “sheep” flavor was preferred by many people in Inner Mongolia, China [7]. 

*A. mongolicum* Regel, a typical herb of the Allium family, grows extensively in the desert steppe of Inner Mongolia, Gansu, and Xinjiang [8]. *A. mongolicum* regel is rich in flavonoids, polysaccharides, essential oils, and other bioactive compounds [9]. Our previous studies have reported that the dietary supplementation of sheep diets with flavonoids from *A. mongolicum* Regel could promote growth performance, as well as improve meat quality and mutton flavor of lambs [10,11]. However, the extraction process of specific active ingredients, such as flavonoids, is complicated, time-consuming, and costly, making it difficult to apply in practical production. *A. mongolicum* Regel ethanol extract (AME) is easier to obtain and less expensive than flavonoids. Ding et al. [12] observed that dietary AME supplementation benefits anti-oxidant status and immune responses without the occurrence of pathological kidney and liver lesions. Liu and Ao [13] found that AME could reduce the deposition of branched-chain fatty acids related to flavor in the subcutaneous and visceral fat depots of lambs to improve mutton flavor. However, no literature currently exists evaluating the effects of AME on the body health status and meat quality of lambs.

We hypothesized that AME would improve the body health status and meat quality of lambs. Therefore, the objective of this study was to investigate the effects of the dietary inclusion of AME on serum index, longissimus dorsi muscle physicochemical characteristics, as well as the amino acid and fatty acid composition of Small-tailed Han sheep.

## 2. Materials and Methods

All experimental procedures involving animals were evaluated and approved according to the guidelines of the Animal Care and Use Committee of Inner Mongolia Agriculture University (Hohhot, China). This study was conducted at a commercial farm in Bayannaoer, Inner Mongolia Autonomous Region, China (latitude 40°13′–42°28′; longitude 105°12′–109°53′) between April and June 2021.

### 2.1. Animals and Experimental Design

A total of thirty 3-month-old male Small-tailed Han sheep (33.60 ± 1.23 kg) were randomly distributed into a control (CON) group and an *A. mongolicum* Regel ethanol extract (AME) group, with 3 replicates in each group and 5 lambs in each replicate. The CON group was offered a basal diet, and the AME group was offered a basal diet with 2.8 g·lamb^−1^·day^−1^ AME. The amount of the AME addition (2.8 g·lamb^−1^·day^−1^) which is most beneficial for lambs was determined by our previous research, [14]. A total of 14 g AME (total dose of 1 replicate, 5 lambs) was weighted to mix concentrate (250 g), divided into two equal amounts, and provided for each replicate twice daily to ensure lambs completely consumed the AME. Each replicate of the CON group was also provided with the concentrate (250 g) without AME in the same manner. The basal diet met the requirements for lambs, as described by NRC (2007) [15], and its composition and nutritional level are shown in Table 1. The trial lasted for 75 d, including a 15 d pre-experiment period and a 60 d feeding period. The lambs were fed twice a day (7:00 and 18:00) and given free access to water.

*A. mongolicum* Regel powder was purchased from Hao Hai Biological Company (Alxa League, Inner Mongolia, China), and the process of extraction was described by Ding [12]. Briefly, the *A. mongolicum* Regel powder was degreased and decolorized using petroleum ether at a ratio of 1:5 (wt/vol) under stirring for 2 h, and then incubated for 24 h. Next, the supernatant was discarded, and the sediment was dried at 65 °C to obtain degreased and decolorized powder. This powder was mixed with ethyl alcohol (75%) at a ratio of 1:5 (wt/vol), and then the mixture was shaken with an ultrasonic cleaner for 15 min. Subsequently, the supernatant remained, and the residue was shaken again following the above steps. The supernatant obtained twice was concentrated using a rotary evaporator, and then the concentrated fluid was freeze-dried to obtain AME. The AME is composed of flavonoids (26.43%), organic acids and their derivatives (18.57%), nucleotides and their derivatives (14.43%), amino acids (11.14%), and other components.

### 2.2. Sampling

At 60 d of the experimental period, a total of 12 lambs, with 2 lambs randomly selected from each replicate, were sampled to collect blood from the jugular vein before the morning feeding using sterile vacuum glass test tubes (5 mL, Huabo Medical Instruments Co., Ltd., Nanjing, China). The blood samples were centrifuged at 3500 rpm/min for 10 min to obtain serum, and then it was stored at −20 °C for serum analyses. After the feeding experiment, the 12 lambs from which blood was collected were fasted overnight and slaughtered. The carcasses were stored at 4 °C for 24 h, and the left side of the longissimus dorsi (LD) muscle between the 7–12th ribs was subsequently separated. The 7–9th rib section of the LD muscle was used to determine amino acid and fatty acid composition. The 9–12th rib section of the LD muscle was used to determine the physicochemical characteristics of the meat.

### 2.3. Chemical Analyses of Feeds

Samples of feed were dried at 55 °C for 48 h and then ground to pass through a 2 mm sieve before chemical analysis. Crude protein (CP, N × 6.25, method 981.10) and mineral (Ca and P) content were measured using an analytical method provided by AOAC [16]. Samples of feeds were tested for neutral detergent fiber (NDF) and acid detergent fiber (ADF) according to the method of Van Soest et al. [17]. The value of NDF and ADF includes the weight of ash.

### 2.4. Serum Analyses

The contents of triglyceride (TG), total cholesterol (TC), high-density lipoprotein (HDL), low-density lipoprotein (LDL), very low-density lipoprotein (VLDL), and non-esterified fatty acid (NEFA) in the serum were measured using a Mindray fully automatic biochemical analyzer (BS-480, Mindray Bio-Medical Electronics Co., Ltd., Shenzhen, China). Leptin (LEP) and insulin (INS) were detected using ELISA kits (Beijing HuaYing Bioengineering Institute, Beijing, China), according to the manufacturer’s instructions, using the Enzyme Labeling Analyzer (DR-200BS, Wuxi HiWell-DiaTek Instruments Co., Ltd., Wuxi, China).

### 2.5. Physical and Chemical Analyses

The pH of three positions (randomly selected) in the LD muscle was measured at 45 min and 24 h post-mortem using a pH-Star machine (pH-Star, Mathaus, Pottmes, Germany), and then the average value was calculated. Buffer solutions of pH 4.00 and pH 7.00 were used to calibrate the pH-Star machine. The colorimeter (SMY-2000SF, SMY Science & Technology Co., Ltd., Beijing, China) was placed directly on the surface of the LD muscle after exposure to oxygen for 50 min to measure meat color, including lightness (L*), redness (a*), and yellowness (b*). Each sample was measured three times, and the mean was determined. Cooking losses (CL) of the LD muscle was determined according to the method described by Honikel [18]. CL (%) = [(sample weight before cooking − sample weight after cooking)/sample weight before cooking] × 100. Next, each sample was cut into patches (1 × 1 × 1 cm) to measure the shear force (SF) of the LD muscle using a tenderometer (C-LM3, Northeastern University, Shengyang, China). Three positions in each patch were selected to measure the SF, and the mean was calculated. The water holding capacity of the LD muscle was determined according to the method described by Cardoso et al. [6]. The moisture, ash, ether extract, and protein were measured according to AOAC [16]. 

### 2.6. Amino Acid Composition Analyses

The LD muscle samples were freeze-dried at −80 °C for 48 h using a lyophilizer (EYELA-FDU-2110, Tokyo Physio Equipment Co., Ltd., Tokyo, Japan). We accurately weighed 0.50 g and 0.20 g of the freeze-dried samples to determine hydrolyzed protein amino acids and free amino acid composition according to the method described by Zhang et al. [19] and Yu et al. [20], respectively, using an automatic amino acid analyzer (L8900, Hitachi, Tokyo, Japan); essential amino acids (EAA) = Thr + Val + Met + Ile + Leu + Phe + Lys + His + Arg; delicious amino acids (DAA) = Asp + Glu +Gly + Ala + Arg + Met.

### 2.7. Fatty Acids Composition Analyses

A total of 0.50 g freeze-dried LD muscle samples were accurately weighed to analyze the fatty acid composition according to the method described by Folch et al. [21] using gas chromatography equipped with flame ionization detection (Clarus680, PerkinElmer, Inc., Waltham, MA, USA) using an SP2560 capillary column (100 m × 0.25 mm × 0.2 μm). Methyl nonadecanoate was used as an internal standard. The injector and detector temperatures were maintained at 220 °C and 260 °C, respectively. The initial oven temperature was programmed at 120 °C for 5 min, and the temperature was then increased to 230 °C at 3 °C/min, held for 5 min, increased to 240 °C at 1.5 °C/min, and held for 13 min, with a split ratio of 1:10. Nitrogen was used as the carrier gas with a flow rate of 1 mL/min.

### 2.8. Statistical Analysis

One-way ANOVA was used to analyze the serum index, meat physicochemical characteristics, amino acid and fatty acid composition data among the treatments using SPSS 26.0 (IBM, New York, NY, USA). The statistical model was Y_i_ = μ + A_i_ + e, where Y_i_ is the dependent variable; μ is the overall mean; A_i_ is the fixed effect of treatment (i = 1.2, CON or AME); and e is the random effect. The *p*-value < 0.05 was defined as statistically significant, and 0.05 < *p*-value < 0.10 was defined as trending toward significance.

## 3. Results

### 3.1. Serum Index

Table 2 reports the serum index levels of the lambs. No significant differences (*p* > 0.05) in the concentration of LDL or VLDL were found between the CON and AME groups. The concentration of TG and TC were significantly decreased (*p* < 0.05), and the concentration of HDL, LEP, and INS were significantly increased (*p* < 0.05) in the AME group compared with the CON group. In addition, AME had a tendency to decrease (0.05 < *p* < 0.10) NEFA levels in the serum.

### 3.2. Meat Physicochemical Characteristics

Table 3 reports the effect of AME on the physicochemical characteristics of the LD muscle. AME supplementation in the diet of lambs had no significant (*p* > 0.05) effect on pH at 45 min, pH 24 h, meat color (L*, a*, b*), or WHC. Compared to the CON group, the SF and CL were significantly decreased (*p* < 0.05) in the AME group. Lambs consuming the AME had a higher (*p* < 0.05) content of ether extract in the LD muscle, but showed no significant difference (*p* > 0.05) in moisture, protein, and ash.

### 3.3. Amino Acid Composition

In this study, we evaluated the effect of AME on hydrolyzed protein amino acids and free amino acids of the LD muscle in lambs. There was no difference (*p* > 0.05) in hydrolyzed protein acids among the treatments (Table 4). In the case of free amino acids (Table 5), a total of 15 amino acids were detected, and neither Ser nor Ala was found. Compared to the CON group, the content of Glu, Gly, TAA, and EAA in the LD muscle were significantly increased (*p* < 0.05) in the AME group. In addition, AME supplementation tended to increase (0.05 < *p* < 0.10) the content of Val, Tyr, and Lys in the LD muscle and tended to decrease (0.05 < *p* < 0.10) Met content.

### 3.4. Fatty Acids Composition

The result of the effect of AME on the fatty acid composition of the LD muscle are presented in Table 6. AME supplementation in the diet of lambs significantly decreased (*p* < 0.05) the content of C18:0 and increased (*p* < 0.05) the content of C16:1 and C22:6 n-3 in the LD muscle. Compared to the CON group, the content of C17:0 tended to decrease, and the content of C18:3 n-3 tended to increase (0.05 < *p* < 0.10) in the AME group. The AME significantly increased (*p* < 0.05) ∑ n-3 PUFAs, and had no effect (*p* < 0.05) on ∑ n-6 PUFAs, resulting in a significant decrease (*p* < 0.05) in n-6:n-3. We also found that AME supplementation decreased (*p* < 0.05) the content of ∑ SFA in the LD muscle, but had no significant effect (*p* < 0.05) on ∑ PUFA:∑ SFA.

## 4. Discussion

### 4.1. Effect of AME on Serum Index of Lambs

In this study, we evaluated the effect of AME on lipid metabolism function, which reflects the growth and development of animals. Our results indicated that AME significantly decreased the concentration of TC and TG, increased HDL content, and had a tendency to reduce NEFA content, which may be attributed to the flavonoids in AME. Our previous study reported that flavonoids from *A. mongolicum* Regel mainly contain rutin, acacetin, quercitrin, 7-O-5,4′-dimethoxy-3′-hydroxide radical flavones, and 3′,4′-epoxygroup-7-O-5-methoxy flavanols [10]. According to our results, Ouyang et al. [22] found that supplementation with mulberry leaf containing flavonoids significantly decreased the content of TC, and TG increased the content of HDL in the serum of Hu sheep. Imai and Nakachi [23] also found that flavonoids decreased the concentration of TC and TG. TG is the product of steatolysis, which can provide energy for various tissues of the body. The lower concentration of TG reflects the higher lipid utilization rate. HDL is involved in the reverse transport of cholesterol from the peripheral tissue to the liver, preventing the occurrence of diseases [24]. Therefore, the higher HDL concentration could accelerate cholesterol metabolism, resulting in low a TC concentration in serum. Previous studies reported that a high concentration of NEFA in the serum could lead to the accumulation of TG in the liver [25]. On the contrary, in this study, AME tended to lower the concentration of NEFA in the serum. Thus, we speculated that AME may reduce the accumulation of TG in the liver and prevent fatty liver formation.

INS inhibits the decomposition of lipids. LEP is a peptide hormone, synthesized and secreted by adipocytes, with multiple functions, which maintains body weight homeostasis and acts as an essential signaling molecule in lipid regulation and food intake [26]. Nagatanid et al. [27] also reported that the subcutaneous injection of LEP in rams can stimulate the secretion of luteinizing and growth hormones. In this study, we observed that the LEP level was significantly increased in the serum of lambs from the AME group, which was probably due to the anti-oxidant and anti-inflammation functions of flavonoids [9] in the AME. Moreover, Haghighatdoost et al. [28] reported that the reduction of inflammation can increase LEP secretion. Overall, AME can regulate the lipid metabolism and maintain the body health of lambs.

### 4.2. Effect of AME on Physicochemical Characteristics of LD Muscle in Lambs

Physicochemical characteristics were used to evaluated the consumer acceptance of the meat. pH is one of the main parameters of meat quality, closely related to the change in meat color. With the cessation of breathing after slaughter, the energy metabolism of the cells transforms from aerobic respiration to anaerobic respiration. Under anaerobic conditions, glycogen is decomposed into lactate by glycolysis, accumulating in the muscle, resulting in the pH change from 7.2 to 5.7 [29]. In this study, we observed no difference in pH 45 min, pH 24 h, and meat color (L*, a*, b*) among the treatments, but these values were within the normal range, which ensures the edible quality of meat. A previous study has observed that feeding lambs with flavonoids from *A. mongolicum* Regel had no effect on pH, which is consistent with our results [11]. The lack of effect of AME on meat color may be attributed to the fact that AME does not affect meat pH.

Muscle contains approximately 75% water, most of which is stored in myofibrils, and a small part is combined with protein. Water losses in the muscle were induced by the shrinkage and denaturation of myofibrils and collagen during cooking, which resulted in the muscles being tighter and harder [30]. Mutton tenderness is also affected by many factors, such as intramuscular collagen content and solubility, intramuscular fat and ultimate pH, sarcomere length, handling before and at slaughter, age, and breed of lambs [31]. The SF is an important index to evaluate the tenderness of mutton. In this study, we observed lower CL and SF and higher intramuscular fat content in the LD muscle of lambs consuming AME. Liu and Ding [11] suggested that dietary flavonoids from *A. mongolicum* Regel supplementation decreased CL and increased intramuscular fat in the LD muscle of lambs. Zhao et al. [32] also found that AME decreased CL and SF and increased intramuscular fat in the LD muscle of Dorper × Small-tailed Han cross-bred sheep, which is consistent with our result. Studies suggested that the content of intramuscular fat is closely related to the concentration of INS in the body [33]. INS is a metabolic hormone that is conducive to adipogenesis and can preferentially promote the utilization of the carbohydrate carbon framework of the intramuscular fat cells to synthesize fatty acids [34]. Therefore, AME improves muscle tenderness, to some extent, through increasing the concentration of INS in the serum to promote the synthesis of intramuscular fat.

### 4.3. Effect of AME on Amino Acids Composition of LD Muscle in Lambs

The amino acid was divided into hydrolyzed protein amino acids and free amino acids. Their distribution in the muscle determines the nutritional value and flavor of mutton [35]. In this study, however, we found that AME did not affect the composition of hydrolyzed protein amino acids of the LD muscle in lambs. Zhao et al. [36] also found that feeding *A. mongolicum* Regel powder to the lambs had no effect on the hydrolyzed protein amino acid composition in the LD muscle of lambs, which is consistent with our results. this may be because hydrolyzed protein amino acids are relatively stable in the muscle. Moreover, studies showed that the protein content of different breeds of sheep and different muscle parts is relatively consistent, and the composition of the diet will not easily change the amino acid composition of mutton, further confirming this view [37].

Some of the free amino acids content in the LD muscle, such as Asp, Gly, Ala, Glu, Phe, and Tyr, was considered as a precursor of flavor compounds, which exhibits a Maillard reaction with soluble reducing sugar to flavor compounds, directly or indirectly affecting the freshness and taste characteristics of meat products [38]. Met is a sulfur-containing amino acid that can produce hydrogen sulfide and flavor compounds through thermal decomposition. Thiophenol was produced by hydrogen sulfide and phenol, which negatively affects the flavor of mutton [39]. In this study, the content of Glu, Gly, TAA, and EAA were significantly increased, the content of Val, Tyr, and Lys tended to be higher, and the content of Met tended to be lower in the LD muscle of lambs in the AME group than in the CON group. Our results suggested that supplementation with AME in the diet of lambs could promote the deposition of free amino acids in mutton, producing a better overall taste in the meat.

### 4.4. Effect of AME on Fatty Acids Composition of LD Muscle in Lambs

The composition and content of fatty acids in meat not only affect consumers’ perception of meat quality, but are also closely related to the occurrence and/or prevention of chronic human diseases [40]. The fatty acid composition of muscle is determined by dietary lipid composition, de novo fatty acid synthesis, desaturation, and differences in the utilization of various fatty acids in animals [41]. Most saturated fatty acids (SFA), such as C14:0 and C16:0, are highly hypercholesterolemic, promoting the accumulation of cholesterol and low-density lipoprotein to increase the risk of developing cardiovascular disease [42]. In contrast, French et al. [43] reported that C16:0 resulted in a reduction in both total and LDL cholesterol when the level of C18:2 exceeded 4.5% of energy in the diet. With regard to C18:0, it is considered neutral, but positively correlated to mutton flavor [44,45]. In addition, Watkins et al. [46] reported that C17:0 was positively correlated with 4-methyloctanoic acid (MOA) and 4-methylnonanoic acid (MNA), the main contributors of “mutton flavor,” and suggested that it could be used as a marker for MOA and MNA. In this study, AME did not affect the concentration of C14:0 and C16:0, but decreased the concentration of C17:0, C18:0, and ∑ SFA in the LD muscle, which may be due to the flavonoids in AME. Previous study reported that flavonoids from *A. mongolicum* Regel could decrease the concentration of C17:0, C18:0, and ∑ SFA in the LD muscle of lambs, which is consistent with our result [11]. Su et al. [5] also found that dietary alfalfa powder supplementation, rich in flavonoids, decreased the C18:0 and ∑ SFA content in the LD muscle of Tibetan sheep.

On the other hand, monounsaturated fatty acids (MUFA) and polyunsaturated fatty acids (PUFA) are considered low-cholesterol fatty acids [47]. In this study, compared with the CON group, the concentration of C16:1, C18:3 n-3, C22:6 n-3,and ∑ n-3 PUFA were significantly increased in the LD muscle in the AME group. C16:1 can effectively regulate glucose and lipid metabolism and has a significant anti-inflammatory activity. Talbot et al. [48] also reported that C16:1 could improve the insulin sensitivity of muscles and counteract the insulin resistance caused by SFA. C18:3 n-3 is an important precursor for the synthesis of C20:5 n-3, C22:6 n-3, and C18:3 n-6 [49]. C20:5 n-3 and C22:6 n-3 can reduce the concentration of triacylglycerol in blood, along with vasodilation and the inflammatory reaction, thereby reducing the risk of cardiovascular disease [50]. The value of ∑ PUFA:∑ SFA and n-6:n-3, above 0.45 and below 4.0, respectively, are widely used to evaluate the value of meat for human health [51]. In this study, although the value of ∑ PUFA:∑ SFA and n-6:n-3 are not within the recommended range, AME significantly decreased n-6:n-3 and had no effect on ∑ PUFA:∑ SFA. However, Realini et al. [52] observed a lower value of ∑ PUFA:∑ SFA (0.13) in mutton. Thus, AME can promote MUFA and PUFA deposit in the muscle, with the potential to improve the mutton odor and flavor of meat.

## 5. Conclusions

In this study, our results indicated that AME dietary supplementation in lambs could affect lipid metabolism. Specifically, AME could increase HDL, LEP, and INS content and decrease TC, TG, and NEFA content in the serum. Meanwhile, AME supplementation could decrease cooking losses and shear force and increase intramuscular fat content in the LD muscle of lambs. In addition, although AME did not affect hydrolyzed protein amino acid composition, it changed the free amino acid composition and promoted the deposition of MUFA and PUFA in the LD muscle of lambs. Therefore, we can consider the use of AME instead of the flavonoids of *A. mongolicum* Regel to improve body health and meat quality in lambs for practical production.

## Figures and Tables

**Table 1:** Composition and nutrient levels of the basal diet (dry matter basis %).

Ingredients	Content (%)
Chinese wildrye	25.00
Caragana	17.80
Whole corn silage	23.60
Wheat bran	5.15
Sunflower seed meal	21.35
Pea stem and leaf	2.64
Red jujube	2.04
CaHPO_4_	0.74
NaCl	0.68
Premix ^a^	1.0
Total	100.00
Nutrient level	
DE ^b^ (MJ/kg)	13.46
CP	16.87
NDF	38.72
ADF	27.51
Ca	1.33
P	0.53

DE = digestible energy; CP = crude protein; NDF = neutral detergent fiber; ADF = acid detergent fiber. Each measurement was conducted with three repetitions. ^a^ Contained 30.00 mg Mn, 25.00 Fe, 29.00 Zn, 8.00 mg Cu, 0.10 mg Co, 0.04 mg I, 3200 IU VA, 1200 IU VD, and 20 IU VE per kilogram premix. ^b^ DE was a calculated value, and others were measured values.

**Table 2:** Effect of AME on the serum index of lambs.

Item	Treatments		SEM	*p*-Value
	CON	AME		
TG, mmol/L	0.85	0.51	0.08	0.017
TC, mmol/L	1.98	1.39	0.16	0.049
HDL, mmol/L	0.86	1.03	0.04	0.015
LDL, mmol/L	0.62	0.63	0.06	0.916
VLDL, mmol/L	0.36	0.42	0.02	0.152
NEFA, mmol/L	0.55	0.31	0.07	0.073
LEP, ng/mL	4.76	6.45	0.29	<0.01
INS, uIU/mL	13.11	14.95	0.39	0.011

CON and AME supplemented with 0 or 2.8 g·lamb^−1^·day^−1^ AME in a basal diet, respectively. Each measurement was performed with three repetitions. TG = triglyceride; TC = total cholesterol; HDL = high-density lipoprotein; LDL = low-density lipoprotein; VLDL = very low-density lipoprotein; NEFA = non-esterified fatty acid; LEP = leptin; INS = insulin.

**Table 3:** Effect of AME on meat physicochemical characteristics of lambs.

Item	Treatments		SEM	*p*-Value
	CON	AME		
pH _45 min_	6.68	6.71	0.07	0.180
pH _24 h_	5.60	5.78	0.06	0.133
L*	37.48	38.83	0.90	0.136
a*	28.03	27.99	0.65	0.230
b*	4.29	5.05	0.62	0.561
CL, %	38.30	34.63	1.02	0.048
SF, kgf	17.01	13.53	0.86	0.035
WHC, %	81.24	84.82	1.16	0.130
Moisture, %	74.86	74.76	1.53	0.380
Protein, %	20.87	21.21	0.92	0.243
Ether extract, %	3.87	5.28	0.76	0.024
Ash, %	1.25	1.32	0.24	0.140

CON and AME supplemented with 0 or 2.8 g·lamb^−1^·day^−1^ AME in the basal diet, respectively. Each measurement was performed with three repetitions. CL = cooking losses; SF = shear force; WHC = water holding capacity.

**Table 4:** Effect of AME on hydrolyzed protein amino acid composition in LD muscle of lambs (fresh sample basis g/100 g).

Item	Treatments		SEM	*p*-Value
	CON	AME		
Asp	1.42	1.32	0.03	0.132
Thr	0.75	0.76	0.02	0.748
Ser	0.62	0.61	0.01	0.736
Glu	2.39	2.40	0.06	0.938
Gly	0.73	0.67	0.01	0.069
Ala	0.96	0.96	0.02	0.875
Cys	0.13	0.12	<0.01	0.169
Val	0.79	0.76	0.02	0.402
Met	0.42	0.40	0.02	0.572
Ile	0.72	0.69	0.02	0.386
Leu	1.34	1.33	0.03	0.872
Tyr	0.56	0.54	0.02	0.577
Phe	0.76	0.70	0.02	0.139
Lys	1.40	1.36	0.03	0.604
His	0.59	0.55	0.02	0.368
Arg	1.00	0.99	0.02	0.770
Pro	0.83	0.84	0.01	0.578
TAA	15.39	15.01	0.31	0.557
EAA	5.60	5.47	0.13	0.644
DAA	5.96	5.79	0.12	0.505

CON and AME supplemented with 0 or 2.8 g·lamb^−1^·day^−1^ AME in the basal diet, respectively. Each measurement was performed with three repetitions. EAA = Thr + Val + Met + Ile + Leu + Phe + Lys + His + Arg; DAA = Asp + Glu +Gly + Ala + Arg + Met.

**Table 5:** Effect of AME on free amino acids composition in LD muscle of lambs (fresh sample basis mg/100 g).

Item	Treatments		SEM	*p*-Value
	CON	AME		
Asp	1.40	1.61	0.08	0.175
Thr	49.81	60.24	3.40	0.129
Glu	7.74	8.75	0.25	0.033
Gly	10.25	12.07	0.43	0.028
Cys	1.33	1.55	0.11	0.348
Val	3.27	3.66	0.11	0.075
Met	1.93	1.57	0.10	0.092
Ile	2.23	2.53	0.09	0.113
Leu	4.41	5.00	0.18	0.105
Tyr	2.45	2.70	0.13	0.077
Phe	5.96	7.08	0.40	0.169
Lys	35.38	39.43	1.16	0.078
His	21.93	22.00	0.44	0.935
Arg	6.62	6.55	0.27	0.911
Pro	30.84	30.74	0.47	0.927
TAA	185.32	205.48	4.45	0.014
EAA	131.53	148.06	4.15	0.039
DAA	27.93	30.56	1.24	0.310

CON and AME supplemented with 0 or 2.8 g·lamb^−1^·day^−1^ AME in the basal diet, respectively. Each measurement was performed with three repetitions. EAA = Thr + Val + Met + Ile + Leu + Phe + Lys + His + Arg; DAA = Asp + Glu +Gly + Ala + Arg + Met.

**Table 6:** Effect of AME on fatty acid composition in LD muscle of lambs (%).

Item	Treatments		SEM	*p*-Value
	CON	AME		
SFAs				
C10:0	0.15	0.20	0.20	0.386
C12:0	0.33	0.20	0.04	0.130
C14:0	2.68	2.38	0.13	0.284
C15:0	0.23	0.20	0.03	0.730
C16:0	22.11	22.30	0.40	0.822
C17:0	0.69	0.63	0.02	0.072
C18:0	16.94	15.07	0.31	0.012
C23:0	2.35	2.36	0.16	0.996
∑SFAs	45.48	43.34	1.04	0.044
MUFAs				
C16:1	0.94	1.71	0.13	<0.01
C17:1	0.38	0.32	0.02	0.159
C18:1 n-9 t	4.49	4.99	0.25	0.339
C18:1 n-9 c	34.39	34.75	0.42	0.690
∑MUFAs	40.20	41.77	0.49	0.136
PUFAs				
C18:2 n-6 t	4.17	3.87	0.38	0.808
C18:2 n-6 c	7.95	8.79	0.49	0.424
C18:3 n-6	0.18	0.19	0.01	0.573
C18:3 n-3	0.21	0.44	0.08	0.073
C20:3 n-3	0.76	0.74	0.23	0.773
C20:5 n-3	0.25	0.28	0.02	0.507
C22:6 n-3	0.16	0.21	0.13	0.033
∑ n-6 PUFAs	12.30	12.85	0.37	0.525
∑ n-3 PUFAs	1.38	1.67	0.06	0.021
∑ PUFAs	13.68	14.52	0.38	0.322
∑ PUFAs:∑ SFAs	0.30	0.34	0.02	0.429
n-6:n-3	8.91	7.69	0.31	0.024

CON and AME supplemented with 0 or 2.8 g·lamb^−1^·day^−1^ AME in a basal diet, respectively. Each measurement was performed with three repetitions.

## Data Availability

The data that support the findings of this study are available from the corresponding author upon reasonable request.
